# Inside-out raising mucosal-tympanomeatal flap approach for the repair of large marginal perforations

**DOI:** 10.1186/s12893-023-02286-y

**Published:** 2023-12-13

**Authors:** Yanting Zhang, Zhengcai Lou

**Affiliations:** 1 Department of operating theater, Yiwu central hospital, 699 jiangdong road, Yiwu city, Zhejiang provice 322000 China; 2 Department of Otorhinolaryngology, Yiwu central hospital, 699 jiangdong road, Yiwu city, Zhejiang provice 322000 China

**Keywords:** Cartilage graft, Inside-out, Mucosal flap, Myringoplasty, Tympanomeatal flap

## Abstract

**Objective:**

This study evaluated the graft success rate and hearing outcomes of the inside-out raising mucosal-tympanomeatal flap technique for the repair of large marginal perforations.

**Study design:**

Prospective case series.

**Materials and methods:**

The study enrolled patients with large marginal perforations who underwent endoscopic cartilage myringoplasty with the inside-out raising mucosal-tympanomeatal flap technique. The graft success rate, hearing outcomes, and complications were evaluated at 12 months postoperatively.

**Results:**

In total, 48 patients with large marginal perforations were included. 81% of the population had large perforation, 14.6 had subtotal and total perforation was seen in 4.2%. The mean operation time was 38.6 ± 7.1 min. At 12 months postoperatively, the graft success rate was 89.6% (43/48). The mean air-bone gap was 25.6 ± 5.2 dB preoperatively and 16.5 ± 4.1 dB at 12 months postoperatively, with significant differences between these values (*p* = 0.001). The functional success rate was 85.4% (41/48). None of the patients experienced worsened sensorineural hearing loss or graft-related complications, such as graft lateralization, significant blunting, and graft medialization, during follow-up.

**Conclusions:**

Endoscopic cartilage-perichondrium myringoplasty for the repair of large marginal perforations using the inside-out raising mucosal-tympanomeatal flap technique was associated with satisfactory graft outcomes and minimal complications.

## Introduction

In general, myringoplasty for repairing large perforations requires raising the mucosal-tympanomeatal flap to reinforce the graft [1, 2]. However, raising the mucosal-tympanomeatal flap and creating a tunnel via an incision in the external auditory canal (EAC) remains technically challenging for novice surgeons. In addition, the process of raising the mucosal-tympanomeatal flap can lead to complications, such as damage to the chorda tympani, EAC stenosis, iatrogenic cholesteatoma, delayed healing, and prolonged operation times [3, 4]. Few studies have been conducted on raising a mucosal-tympanomeatal flap for repairing anterior marginal perforations [5–7]. To date, there has been minimal discussion regarding the technique of raising a mucosal-tympanomeatal flap without the need for additional EAC incisions when treating large marginal perforations. The aim of this study was to evaluate the graft success and hearing outcomes associated with the cartilage-perichondrium graft technique, which involves inside-out elevation of the mucosal-tympanomeatal flap without additional EAC incisions for the repair of large marginal perforations.

## Materials and methods

### Ethical considerations

The study protocol was approved by the Institutional Ethical Review Board of Yiwu Central Hospital, Yiwu, Zhejiang, China (approval date: July 21, 2020; approval no.: K2020-IRB-027). Informed consent was obtained from all participants.

### Study design

This prospective case series enrolled consecutive patients with chronic large marginal perforations who underwent the cartilage-perichondrium graft technique with inside-out elevation of a mucosal-tympanomeatal flap. The inclusion criteria were as follows: marginal perforations with the absence of tympanic membrane (TM) remnant on at least one side of the perforation; large perforations covering more than one-fourth of the TM area; patient age of 18–75 years; involvement of the malleus handle; dry ear for at least 3 months prior to surgery; a positive Valsalva maneuver; sensorineural hearing loss with a need for TM reconstruction to prevent middle ear infection due to water entry into the ear. We excluded patients with a need for revision surgery, a need for ossicular chain reconstruction, middle ear cholesteatoma or inflammation, or fungal otitis externa. Marginal perforation was defined as the absence of any TM remnant on at least one side before surgery or after de-epithelializing the margins. Perforations were categorized as large (covering one-fourth to half of the TM area), subtotal (half to three-fourth of the TM area), and total (more than three-fourths of the TM area). All surgeries were performed by the same surgeon. All patients underwent temporal bone high-resolution computed tomography preoperatively. Operative time was determined from graft harvesting to EAC packing.

Audiometric data were collected before and 12 months after surgery. Pure-tone averages (PTAs) were determined using the findings at 500, 1,000, 2,000, and 3,000 Hz for both air conduction and bone conduction. For most patients, the threshold at 4,000 Hz was used to interpolate the threshold at 3,000 Hz, following the recommendations of the Hearing Committee of the American Academy of Otolaryngology–Head and Neck Surgery. Pre- and postoperative air-bone gaps (ABGs) were calculated by subtracting air-conduction PTAs from bone-conduction PTAs. ABG closure was determined by subtracting the preoperative ABG from the postoperative ABG.

### Surgical techniques

The patient was positioned in the supine position with the head inclined upward at 30°, facing the opposite side of the surgeon. Video equipment with a camera system (Hangzhou Tonglu Endoscope Co., Ltd., Hangzhou, China) was placed on the opposite side of the surgeon. All patients underwent myringoplasty with a cartilage-perichondrium graft using a 0° rigid endoscope (Hangzhou Tonglu Endoscope Co., Ltd.) under total intravenous anesthesia.

We developed an inside-out approach for cases where no TM remnant exists, eliminating the need to raise a mucosal-tympanomeatal flap through an EAC incision. The medial EAC skin and annulus were elevated, beginning approximately 2 mm away from the mucosa of the tympanic sulcus using an inside-out approach to raise the mucosal-tympanomeatal flap at least 2 mm off the bone. This approach is also referred to as antidromic raising of the mucosal-tympanomeatal flap. This procedure only involved the use of a microscopic cross-cutting knife and did not require any other techniques or surgical tools.

**Fig. 1:**
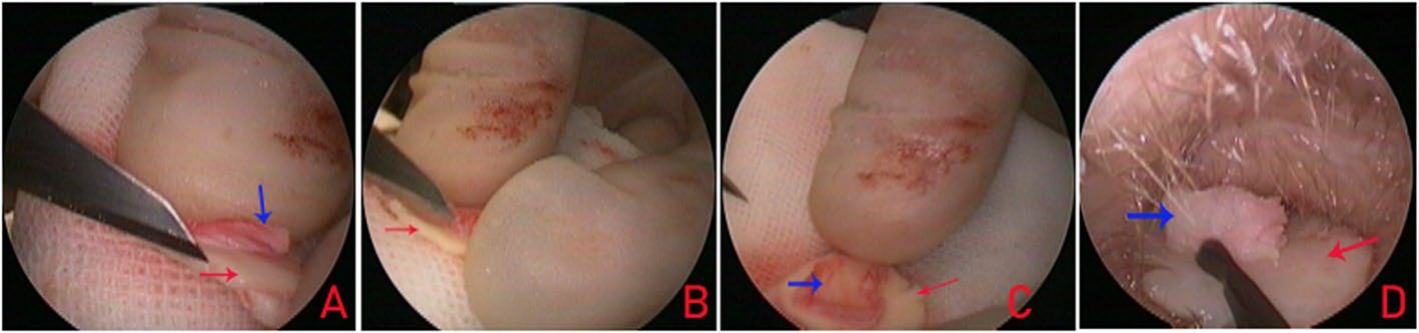
Preparation of cartilage perichondrium. **A**–**C** Perichondrium elevation; (**D**) placement of cartilage perichondrium graft

Cartilage, consisting of a one-sided perichondrium graft, was harvested from the ipsilateral tragus. The cartilage, along with the perichondrium, was harvested to match the perforation size. The perichondrium was elevated circumferentially, with its pedicle attached to the cartilage. The cartilage was approximately 1 mm larger than the perforation margins (Fig. 1), whereas the perichondrium was at least 2 mm larger than the cartilage graft. Additionally, a notch was created in the cartilage graft to accommodate the malleus handle.

**Fig. 2:**
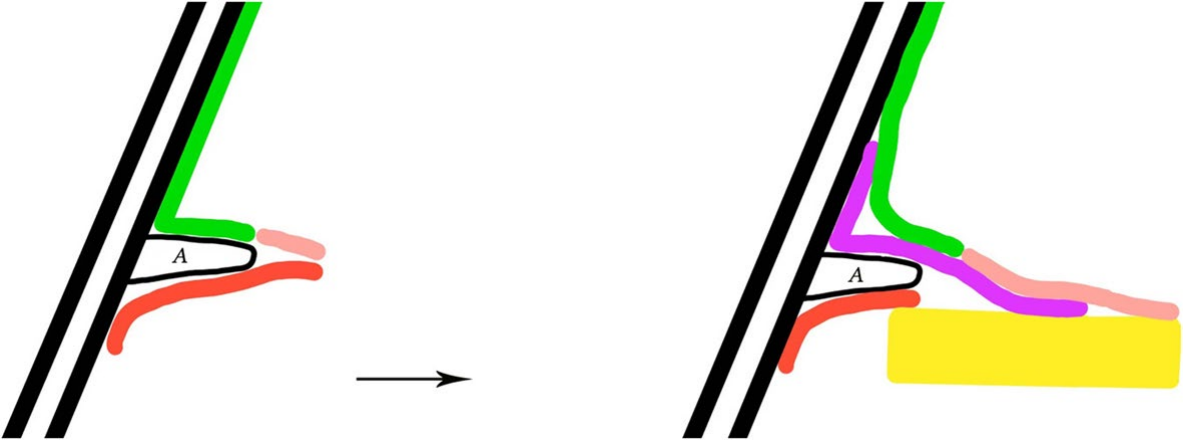
Illustration of the surgical process. Black: external auditory canal bone; Green: EAC skin; Pink: TM remnant; Red: mucosal layer; Yellow: cartilage graft; Purple: perichondrium graft; A: bone annulus

**Fig. 3:**
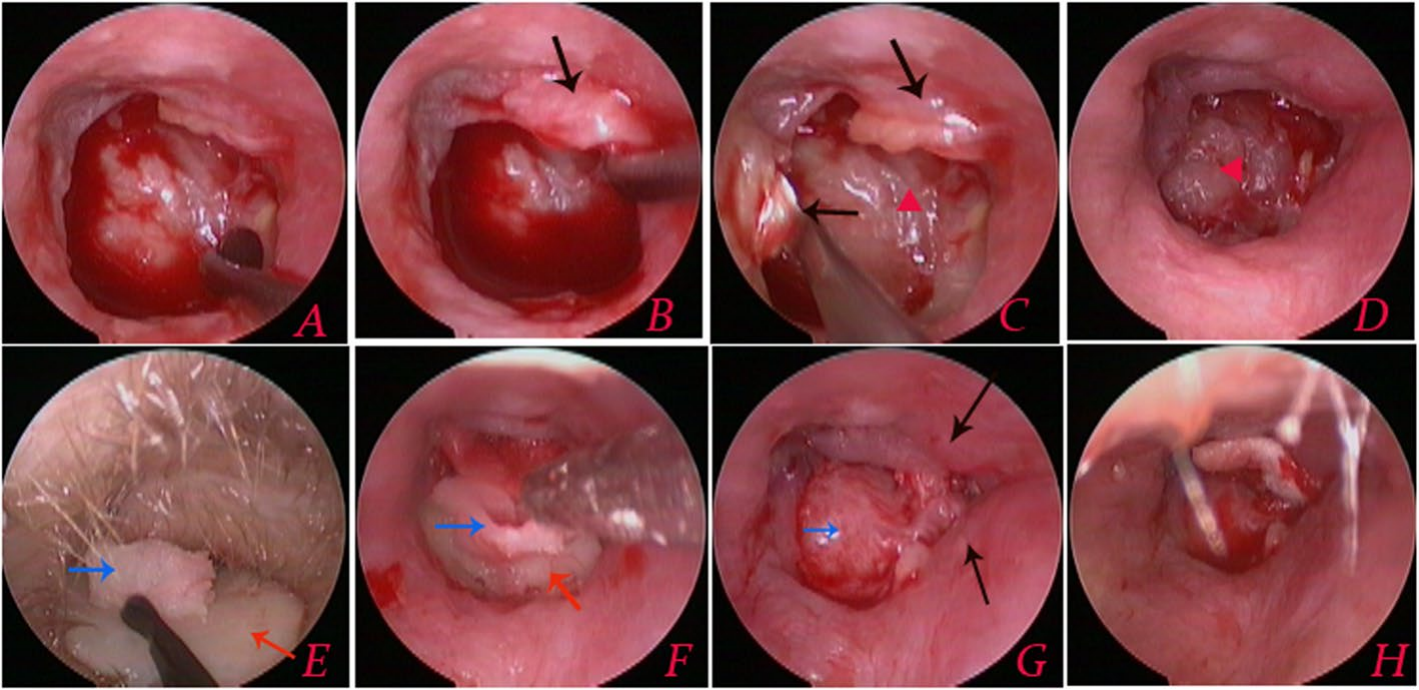
**A** Preoperative image of the perforation; (**B**–**D**) flap elevation involving the middle ear mucosa and EAC skin without an additional EAC incision; (**E**, **F**) placement of the cartilage-perichondrium graft; (**G**) the cartilage was positioned medially to the TM remnant, with the perichondrium graft placed beneath the flap; and (**H**) lateral packing of the graft. Black arrows indicate the mucosal-tympanomeatal flap; red arrows indicate the cartilage; blue arrows indicate the perichondrium; and red triangle indicates the bone forming the medial wall of the middle ear

The perforation edges were de-epithelialized, and the mucosal-tympanomeatal flap was elevated, beginning approximately 2 mm away from the mucosa of the tympanic sulcus using a microscopic cross-cutting knife (Figs. 2 and 3). This was necessary in cases where the TM remnant was insufficient to secure the graft. Then, the fibrous annulus and medial EAC skin were elevated using an inside-out approach, ensuring they were at least 2 mm away from the bone to create the mucosal-tympanomeatal flap. The epithelium was removed from the distal malleus handle.

The middle ear was packed tightly to the level of the perforation using biodegradable synthetic polyurethane foam packing (Stryker Canada, Hamilton, Canada) that had been soaked in antibiotic ointment. The perichondrium-cartilage composite graft was inserted trans-perforation, with the cartilage graft placed medial to the TM remnant and bone annulus. The notch in the graft accommodated the malleus, whereas the perichondrium graft was positioned lateral to the malleus handle and bone annulus, tucked inside the mucosal-tympanomeatal flap (Fig. 2). The EAC was initially filled with biodegradable synthetic polyurethane foam packing. Then, the cartilaginous part was packed with antibiotic-soaked gauze, extending up to the tragus incision.

### Postoperative follow-up

Patients were discharged on the day following the surgery. All patients received a course of antibiotics (amoxicillin) postoperatively. Postoperative follow-up appointments were scheduled in the outpatient department at 2 and 4 weeks and 3 and 12 months. At each follow-up visit, endoscopic examination was performed to assess the graft status. Patients were also asked about altered taste, vertigo, and tinnitus. A postoperative ABG of ≤ 20 dB was considered a functional successful outcome. Graft success was defined as an intact graft without perforation, retraction, lateralization, significant blunting, or medialization.

### Statistical analyses

Statistical analyses were performed using SPSS software (version 20; IBM Corp., Armonk, NY, USA). Data are expressed as means with standard deviations or numbers with percentage. Differences between preoperative and postoperative ABGs were analyzed using the paired-samples *t*-test. *P*-values < 0.05 were considered indicative of statistical significance.

## Results

### Baseline characteristics

In total, 48 patients (31 [64.6%] females and 17 [35.4%] males; mean age: 52.3 ± 8.6 years) with a unilateral large marginal perforation were included in the study. The perforation affected the left ear in 21 (43.8%) patients and the right ear in 27 (56.2%) patients. The mean duration of perforation was 19.7 ± 5.1 years. Of the 48 patients, 81%, 14.6%, and 4.2% had large, subtotal, and total perforations, respectively. Sensorineural hearing loss was absent in 45 (93.8%) patients, whereas 3 (6.2%) patients aged > 65 years had sensorineural hearing loss. The mean operation time was 38.6 ± 7.1 min.

### Graft success and hearing gain

**Fig. 4:**
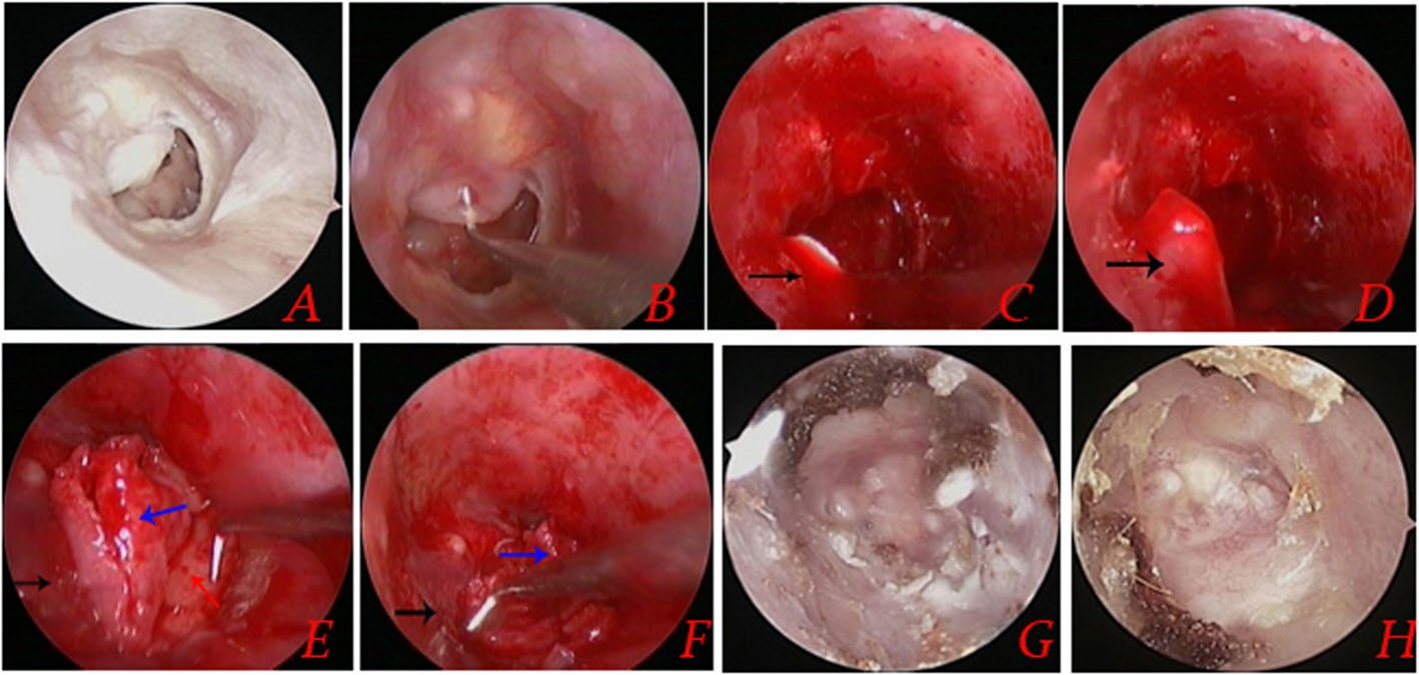
**A **Preoperative image of the perforation; (**B**) de-epithelialization of the perforation margin; (**C**, **D**) flap elevation; (**E**, **F**) placement of the cartilage-perichondrium graft; (**G**) 4 weeks postoperatively; and (**H**) 2 months postoperatively. Red arrows indicate inadequate TM remnant; black arrows indicate the mucosal-tympanomeatal flap; red arrows indicate the cartilage; and blue arrows indicate the perichondrium

Postoperative middle ear infection developed in 2 (4.2%) patients, leading to residual perforation at 1 month postoperatively. Furthermore, a small residual perforation was observed in 3 (6.3%) patients without middle ear infection at 3 months postoperatively, whereas no cases of residual perforation or re-perforation were observed at 12 months postoperatively. The graft success rates were 95.8%, 89.6%, and 89.6% (43/48) at 1, 3, and 12 months postoperatively. Figure 4 presents the surgical procedure and postoperative healing.

Audiological testing was performed in 48 patients at 12 months postoperatively, which showed no change in the sensorineural threshold. The mean ABG was 25.6 ± 5.2 dB preoperatively and 16.5 ± 4.1 dB at 12 months postoperatively, with significant differences between these values (paired-samples *t*-test *p* = 0.001). The ABG was < 10 dB in 11 (22.9%) patients, 10–20 dB in 30 (62.5%), and > 20 dB in 7 (14.6%), corresponding with a functional success rate of 85.4% (41/48).

### Complications

None of the patients developed worsening sensorineural hearing loss, altered taste, facial nerve palsy, vertigo, tinnitus, or graft-related complications, such as graft lateralization, significant blunting, or graft medialization, during follow-up.

## Discussion

During myringoplasty, it is challenging to repair large marginal perforations, which typically require raising a mucosal-tympanomeatal flap by making an incision in the EAC to reinforce the graft [1, 2, 8]. However, raising the mucosal-tympanomeatal flap via an EAC incision is time-consuming and can lead to chorda tympani damage, EAC stenosis, iatrogenic cholesteatoma, and delayed healing [3, 4, 9].

The technique of elevating the medial ear canal skin anteriorly has been used to repair anterior perforations [5–7]. Bluher et al. [5] described the “back-elevation” of the ‘‘window shade’’ technique for repairing anterior marginal perforations. This technique involves elevating a laterally-based anterior canal wall skin flap, beginning at the anterior fibrous annulus. The investigators reported a graft success rate of 94.2% [5].

Gülşen et al. [6, 10] elevated the mucosal flap without additional anterior canal wall incisions to repair anterior perforations, achieving a success rate of 93.7%. Their aim was to improve postoperative vascular supply to the anterior graft and reduce the amount of exposed bone compared to the traditional skin-graft-based lateral tympanoplasty technique. In a previous study, we used the inside-out elevation of a mucosal-tympanomeatal flap to repair anterior perforations, achieving a success rate of 92.2% at 6 months postoperatively [7].

Unlike previous studies, [5, 6, 10] we did not perform additional EAC skin incisions for the repair of anterior perforations. The previously described techniques require a mucosal-tympanomeatal flap via an EAC skin incision combined with raising a mucosal flap [5, 6, 10]. The use of a mucosal-tympanomeatal flap via an EAC skin incision is associated with increased risks of wound infection and the formation of epithelial pearls [10, 11]. Gülşen et al. [10] found that wound infections developed in 3 (8.5%) patients. Schraff et al. [11] revealed the formation of epithelial pearls in 2 (22%) patients and wound infection in 1 (11%) out of 9 patients.

Conventional endoscopic cartilage tympanoplasty typically involves raising a mucosal-tympanomeatal flap via an EAC skin incision to repair large marginal perforations [1, 2, 8]. However, few studies have been conducted on raising a mucosal-tympanomeatal flap without an EAC skin incision to repair large marginal perforations. In this study, we described an inside-out technique for raising a mucosal-tympanomeatal flap without the need for an additional EAC incision to repair large marginal perforations. Notably, the direction in which the mucosal-tympanomeatal flap was created (i.e., anterior, posterior, or inferior EAC) did not significantly affect the procedural complexity. Subsequently, the cartilage graft was positioned medial to the TM remnant and bone annulus, with the perichondrium graft extending onto the medial bony annulus and EAC, tucked beneath the mucosal-tympanomeatal flap. This approach strengthened the perichondrium graft, which was secured to the cartilage using the pedicle attachment, thereby increasing graft stabilization, preventing graft displacement, and improving the graft success rate.

In the present study, although 4.2% of patients had a residual perforation with postoperative middle ear infection and 6.3% had a residual small perforation without middle ear infection, the graft success rate was 89.6% (43/48) at 12 months postoperatively. Our results demonstrated successful repair of large marginal perforations using the antidromic raising mucosal-tympanomeatal flap technique, with no need for additional EAC skin incisions. The graft success rate achieved with this technique is comparable to that of other techniques for the repair of large marginal perforations that require raising a mucosal-tympanomeatal flap via an EAC skin incision. Shoman et al. [12] performed modified palisade cartilage myringoplasty along with elevation of medial ear canal skin to repair subtotal perforations, achieving a success rate of 77%. Angeli et al. [13] demonstrated a success rate of 91% with the lateral technique. Cohen-Vaizer et al. [14] performed inlay triple-“C” tympanoplasty to repair large marginal perforations and obtained a success rate of 90.8%. Casas et al. [15] applied the L-shaped cartilage graft technique and classical underlay technique for subtotal and total perforations, achieving graft success rates of 94.4% and 78.3%, respectively. Dhungana et al. [16] achieved a graft success rate of 90.3% (28/31) using the “U” flap technique, whereas other investigators reported a success rate of 89.8% using the anterior tab flap technique [17] and 91% using the lateral graft technique [13].

The primary concerns in this study were the operation time and complications. The mean operation time was 38.6 ± 7.1 min, which was significantly shorter than other techniques involving the raising of a tympanomeatal flap via an EAC incision. Plodpai et al. [9] demonstrated a mean operating time of 101.6 min using the overlay technique with tympanomeatal flap elevation, whereas Chen et al. [18] reported a mean operating time of 99.9 ± 26.7 min and Atchariyasathian et al. [19] revealed a mean operating time of 100 ± 28 min for endoscopic type I tympanoplasty. In the present study, there were significant differences between the mean preoperative and postoperative ABGs. Notably, 6.2% patients aged > 65 years had ossicular chain abnormalities without the need for ossicular reconstruction. However, this technique is unlikely to lead to EAC stenosis due to the absence of an EAC incision. None of the patients developed worsened sensorineural hearing loss, altered taste, facial nerve palsy, or graft-related complications, including graft lateralization, significant blunting, and graft medialization, during follow-up. The present approach can also be used during microscopic graft techniques. However, this study had certain limitations. First, this study had a small sample size and short follow-up duration. Second, this study did not include a control group. Future studies should include long-term follow-up and a control group. Third, none of the patients in the present study developed altered taste or damage to the chorda tympani, which might be attributed to the smaller number of patients with posterior superior marginal perforation. Furthermore, there seems to be a potential risk of nerve and ossicle damage when performing inside-out mucosal elevation for these patients. Therefore, future studies should include a larger population with posterior superior marginal perforation.

Fourth, we did not evaluate the relationships between graft success, functional success (ABG), and perforation size due to the unequal number of patients with perforations of different sizes (i.e., large, subtotal, and total).

## Conclusions

Endoscopic cartilage-perichondrium myringoplasty with inside-out elevation of mucosal-tympanomeatal flap is associated with satisfactory graft outcomes and minimal complications during the repair of large marginal perforations.

## Data Availability

All data generated or analyzed during this study are included in the published manuscript.

## References

[1] Nicholas Jungbauer W, Jeong S, Nguyen SA, Lambert PR (2023). Comparing myringoplasty to type I tympanoplasty in tympanic membrane repair: a systematic review and meta-analysis. Otolaryngol Head Neck Surg.

[2] Furukawa T, Ito T, Kubota T, Futai K, Matsui H, Kakehata S (2022). The feasibility and treatment results of transcanal endoscopic myringoplasty. Otol Neurotol.

[3] Berglund M, Suneson P, Florentzson R (2019). Tinnitus and taste disturbances reported after myringoplasty: data from a national quality registry. Laryngoscope.

[4] Takihata S, Kurioka T, Mizutari K, Shiotani A (2022). Factors affecting the incidence of chorda tympani nerve transection in middle ear Surgery. Laryngoscope Investig Otolaryngol.

[5] Bluher AE, Mannino EA, Strasnick B (2019). Longitudinal analysis of window shade tympanoplasty outcomes for anterior marginal tympanic membrane perforations. Otol Neurotol.

[6] Gülşen S, Arıcı M (2020). Reply to the letter to the editor concerning ‘The of elevation of the mucosal flap without additional anterior canal wall incisions for repairing anterior perforations using endoscopic cartilage tympanoplasty’. Eur Arch Otorhinolaryngol.

[7] Lou Z (2020). Endoscopic cartilage myringoplasty with inside out elevation of a tympanomeatal flap for repairing anterior tympanic membrane perforations. Ann Otol Rhinol Laryngol.

[8] Takahashi M, Motegi M, Yamamoto K, Yamamoto Y, Kojima H (2022). Endoscopic tympanoplasty type I using interlay technique. J Otolaryngol Head Neck Surg.

[9] Plodpai Y (2018). Endoscopic vs microscopic overlay tympanoplasty for correcting large tympanic membrane perforations: a randomized clinical trial. Otolaryngol Head Neck Surg.

[10] Gülşen S, Arıcı M (2019). Endoscopic transcanal versus conventional microscopic tympanoplasty in treatment of anterior tympanic membrane perforations. Eur Arch Otorhinolaryngol.

[11] Schraff S, Dash N, Strasnick B (2005). Window shade tympanoplasty for anterior marginal perforations. Laryngoscope.

[12] Shoman NM (2019). Clinical and audiometric outcomes of palisade cartilage myringoplasty under local anesthetic in an office setting. Am J Otolaryngol.

[13] Angeli SI, Kulak JL, Guzmán J (2006). Lateral tympanoplasty for total or near-total perforation: prognostic factors. Laryngoscope.

[14] Cohen-Vaizer M, Barzilai R, Shinnawi S (2021). Inlay triple- C tympanoplasty: a comparative study for its use in large, marginal perforations. Eur Arch Otorhinolaryngol.

[15] Linares Casas A, Ruiz R, De Pauli D (2022). Endoscopic type 1 tympanoplasty; a composite graft technique for subtotal and total perforations. Eur Arch Otorhinolaryngol.

[16] Dhungana S, Rayamajhi P, Shrivastav RP (2018). Outcome of graft uptake and hearing results between U flap technique and conventional tympanomeatal flap technique for anterior and subtotal tympanic membrane perforation. J Nepal Health Res Counc.

[17] Faramarzi M, Atashi S, Edalatkhah M, Roosta S (2021). The effect of anterior tab flap technique on graft success rate in large tympanic membrane perforation. Eur Arch Otorhinolaryngol.

[18] Chen CK, Hsu HC, Wang M (2022). Endoscopic tympanoplasty with post-conchal perichondrium in repairing large-sized eardrum perforations. Eur Arch Otorhinolaryngol.

[19] Atchariyasathian V, Suwannajak R, Plodpai Y, Pitathawatchai P (2020). A comparison of endoscopic transtympanic myringoplasty and endoscopic type I tympanoplasty for repairing medium- to large-sized tympanic membrane perforation: a randomized clinical trial. Eur Arch Otorhinolaryngol.

